# Copper Electroless Metallization of Cellulose Paper via Polydopamine Coating and Silver Catalyst

**DOI:** 10.3390/ma14226862

**Published:** 2021-11-14

**Authors:** Krzysztof Moraczewski, Andrzej Trafarski, Rafał Malinowski

**Affiliations:** 1 Institute of Materials Engineering, Kazimierz Wielki University, Chodkiewicza 30, 85-064 Bydgoszcz, Poland; trafarski@ukw.edu.pl; 2 Łukasiewicz Research Network-Institute for Engineering of Polymer Materials and Dyes, Marii Skłodowskiej-Curie 55, 87-100 Toruń, Poland; malinowskirafal@gmail.com

**Keywords:** flexible electronics, cellulose paper, polydopamine, copper electroless metallization

## Abstract

The paper presents the results of copper electroless metallization of cellulose paper with the use of a polydopamine coating and silver catalyst. The polydopamine coating was deposited via a simple dip method using a dopamine hydrochloride solution in 10 mM TRIS-HCl buffer with a pH of 8.5. The research showed that as a result of this process, cellulose fibers were covered with a homogeneous layer of polydopamine. The unique properties of the polydopamine coating allowed the reduction of silver ions from silver nitrate solution and the deposition of silver atoms on the paper surface. Deposited silver served as a catalyst in the autocatalytic electroless copper-plating process. The copper layer covered the entire surface of the paper sheet after 5 min of metallization, favorably affecting the electrical properties of this material by lowering the surface resistivity. The deposited copper layer was further characterized by good adhesive strength and high susceptibility to deformation.

## 1. Introduction

Cellulose is a well-known natural polymer that has many advantages such as: low price, renewability, ease of processing, biodegradability and good mechanical properties. Cellulose has no taste, is odorless, is hydrophilic, has a contact angle of 20–30 degrees, and is insoluble in water and most organic solvents [1]. Many properties of cellulose depend on its chain length or degree of polymerization, the number of glucose units that make up one polymer molecule. Cellulose from wood pulp has typical chain lengths between 300 and 1700 units; cotton and other plant fibers as well as bacterial cellulose have chain lengths ranging from 800 to 10,000 units [2].

As the main constituent of wood and plants, cellulose is an almost inexhaustible polymeric raw material on the earth. It has a fascinating structure and is highly biocompatible and easily biodegradable. It was reported that the elastic modulus of the crystalline region of cellulose might reach the level as high as 100–200 GPa, and a density as low as 1.6 g/cm^3^. In particular, due to possessing large amounts of hydroxyl groups, cellulose is a good object for chemical modification, which endows it with a wider range of applications, such as being used as reinforcement, and as a thickener, emulsifier and stabilizer [3].

Cellulose is mainly used to produce paperboard and paper. Smaller quantities are converted into a wide variety of derivative products such as cellophane and rayon. It is also used in the production of textiles, hygiene products and pharmaceuticals. The conversion of cellulose from energy crops into biofuels such as cellulosic ethanol is under development for the provision of renewable fuel sources. Cellulose for industrial use is mainly obtained from wood pulp and cotton [4,5,6].

Of all the cellulose products, paper is perhaps the most widely produced and used [2,7]. Paper plays a significant role in writing, printing, packaging, currency, origami, personal hygiene, medicine and construction. These applications are possible through a diverse set of manufacturing processes and chemical additives. Modification of the fibrous matrix of paper can tune its hydrophilicity or hydrophobicity, porosity, opacity or transparency, and surface roughness [8,9,10].

In addition to its common and well-known uses, in recent years, paper has attracted a lot of attention as a material for advanced electronics applications. By means of various techniques and methods, paper can be transformed into an excellent material for the construction of various electronic devices. Due to the mentioned properties and with appropriate treatment, cellulose paper can be used as flexible/transparent substrates, separators, electronic–ionic conductors, electrolytes and electrochemical electrode materials in flexible circuits or sensors, conductive transistors, organic light-emitting diodes (OLEDs), organic thin-film transistors (OTFTs), supercapacitors, batteries, triboelectric nanogenerators (TENGs), tissue bioelectronics and other flexible electronics [11,12,13,14,15,16,17]. The area of flexible paper-based electronic devices in particular has recently been an area of intense research.

As previously mentioned, in order for the paper to be used in advanced electronic applications, it requires specialized treatment. In recent years, a number of chemical or physical methods and techniques have been used for this purpose, which can be divided into the following categories: electronics on paper and electronics in paper [18]. The first includes, among others, electroless deposition, spray-coating, evaporative deposition, sputtering, flexographic printing, microfluidic spinning, electrostatic spinning and various methods of 3D printing [18,19,20]. The second is related to the introduction into the paper volume of components that change electrical properties, i.e., metallized particles or inorganic or organic fibers, such as mica, glass microspheres, glass fibers, polymer fibers, as well as fillers such as graphite or metal particles [21,22,23].

Among the mentioned methods, the electroless deposition, namely its autocatalytic variant, deserves special interest. It is a well-known, simple and widely used method for metallizing non-conductive materials, e.g., polymers, glass or paper [24,25,26]. In the autocatalytic deposition of a metal layer, the metal ions in the metallization bath are reduced in the presence of a catalyst. The reduction reaction begins when the bath is in direct contact with the catalyst and only occurs at the surface of the catalyst. The catalyst is the metal from which the covered product is produced or another metal deposited on the surface of the product. A prerequisite is that the reduction reaction is catalyzed by the deposited metal, because this is why it proceeds autocatalytically after covering the substrate and is, therefore, called autocatalytic deposition [27]. Apart from its numerous advantages, this method has some disadvantages related to environmental issues, a relatively high cost of catalysts (mainly palladium compounds) and a long deposition time. Therefore, research is carried out to eliminate these unfavorable features of this process.

One of the more interesting solutions that are an alternative to electroless deposition with the use of a palladium catalyst is the use of biomimic chemical compounds, which was inspired by the animal world. In 2007, Lee et al. [28] presented a method for the preparation of multifunctional polydopamine coatings formed in the dopamine autopolymerization process. Their research was inspired by observations of mussels. These organisms show great adhesion to underwater structures. Mussels can adhere with great force to virtually all inorganic and organic surfaces, including hard-to-stick materials such as poly(tetrafluoroethylene) (PTFE). The mussels owe their strong adhesion abilities due to the produced chemical compounds from the group of protein amino acids, such as catecholamine 3,4-dihydroxy-L-phenylalanine (DOPA) and amino acids of lysine [29]. Although it is mainly catecholamines and lysine amino acids that are responsible for the good adhesion of mussels to a wide range of surfaces, it has been shown that dopamine, a derivative of DOPA, also has similar properties.

Dopamine is an organic chemical compound from the catecholamine group [30]. It is an important neurotransmitter synthesized and released by neurons of the central nervous system. The proper implementation of the dopamine polymerization process allows the obtaining of a thin film of polydopamine coating on virtually any type of substrate. The process of polydopamine coating deposition is simple and effective. It consists in immersing the coated product in an aqueous solution of dopamine, buffered to a pH typical of the marine environment (pH = 8.5), typically at room temperature. The result of this process is the spontaneous formation of a strongly adhering coating of polydopamine. The characteristic feature and great advantage of the polydopamine coating is its high reactivity. A very impressive feature is the ability of polydopamine coating to reduce metallic cations by direct contact of the film with an aqueous solution [31,32]. Metallic nanoparticles are known for their catalytic activity owing to their high surface area. The fact that metal nanoparticles can be directly deposited on polydopamine films from a metal cation solution, without the need to graft already synthesized nanoparticles on the substrate, offer fascinating perspectives for catalysis that can be used in the electroless metallization of various materials [33,34,35,36,37].

The aim of the research presented in the article was to test the possibility of using a polydopamine coating in the process of electroless metallization of cellulose paper. The research combined two research areas that have recently been of great interest, namely flexible electronic devices based on paper and biomimic methods of surface modification of engineering materials. Thanks to the unique properties of the paper and the advantages of using a polydopamine coating, it will be possible to obtain excellent flexible electronic devices in a process that will be faster, cheaper and more environmentally friendly compared to the classic method of electroless metallization, as well as other chemical or physical methods of metallizing paper.

## 2. Materials and Methods

### 2.1. Materials

The retail POL Copy paper (International Paper, Poland) with a grammage of 80 g/m^2^ was metallized. Dopamine hydrochloride C_8_H_12_ClNO_2_ (molecular weight 189.64 g/mol) (Sigma Aldrich, Poznań, Polska) and 10 mM TRIS-HCl buffer (Chempur, Piekary Śląskie, Poland) with a pH of 8.5 were used in the modification process. Silver nitrate AgNO_3_ (pure, molecular weight 169.88 g/mol) (Sigma Aldrich, Poznań, Poland) was used in the surface activation process (deposition of catalyst). The metallization was performed in a commercial six-component M-Copper 85 electroless copper-plating bath (MacDermid, Waterbury, CT, USA).

### 2.2. Methodology

**Figure 1:**
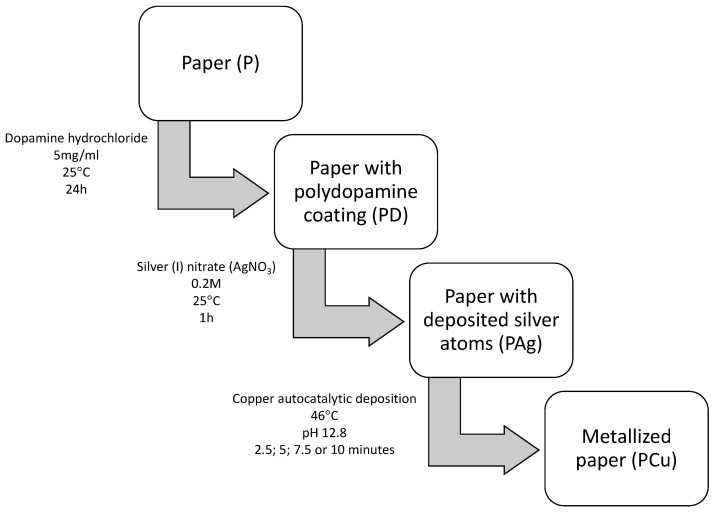
Flow diagram of paper metallization process.

The process of depositing the polydopamine coating on the paper sheets was carried out without prior preparation (Figure 1). The polydopamine coating deposition solution consisted of 5 mg/mL dopamine hydrochloride dissolved in TRIS-HCl buffer [28]. Sheets of paper were placed in the deposition solution and soaked for 24 h at a temperature of 25 °C. The sheets were then rinsed with distilled water and dried for 24 h. The material obtained in this way is designated as PD in further tests.

Sheets with deposited polydopamine coating were immersed in an aqueous solution of silver (I) nitrate (AgNO_3_) at a concentration of 0.2 M in order to activate the surface [38]. The activation process was carried out for 1 h at 25 °C. The sheets were then rinsed with distilled water and dried for 24 h. The material obtained in this way is designated as PAg in further tests.

The activated samples were consequently immersed in commercial autocatalytic M-Copper 85 (MacDermid, Waterbury, CT, USA) copper-plating bath, with formaldehyde as a reducing agent, at 46 °C. The pH value of the bath at a temperature of 46 °C was 12.8. Following the recommendations of the manufacturer, during the metallization process, the bath was continuously aerated. The samples were metallized for 2.5; 5; 7.5 or 10 min. In the post-treatment stage, the samples where dried in a laboratory oven. The metallized samples were designated as PCu2_5; PCu5, PCu7_5 and PCu10 as a function of the metallization time.

The microscopic images of the sample surfaces were taken with a LEICA DMS-350 digital microscope (Leica Microsystems, Heerbrugg, Switzerland), with Leica Application Suite LAS 4.12 image processing software.

The surface of the samples was observed at higher magnifications using a Phenom XL (Thermo Fisher Scientific, Waltham, MA, USA) scanning microscope with an EDS attachment designed for chemical composition analysis. Depending on the sample, the observations of their surfaces on the SEM microscope and EDS analysis were carried out at various magnification levels from 245× to 5200×, with accelerating voltages from 10 kV to 15 kV, in the mapping mode, in the BSD Full (Back Scatter Detector) backscattered electron detector, with a high vacuum of 0.1 Pa. The total size of the observation area FOV (field of view) ranged from 51.6 µm to 955 µm and the working distance ranged from 7.6 mm to 8.3 mm. Before imaging, the surface of the tested samples was covered with a layer of gold. The conductive layer was sputtered in a MCM100P (SEC, Suwon-si, Korea) low-vacuum sputtering machine for 2 min.

Volume and surface resistivity measurements were made using a set of instruments including a Model 6517A electrometer and a Model 8009 electrode array (Keithley Instruments Inc., Cleveland, OH, USA). During the measurements of the volume resistivity and surface resistivity, the polarization voltage was 500 V, and its duration was 30 s. The average of 10 measurements of the current intensity was taken as the result.

Adhesive strength was determined by the non-standard method, aimed only at determining this parameter in the range of good/bad adhesive strength without specifying specific values. In order to determine the adhesive strength, the Perfect Extra Power adhesive tape (Tesa SE, Norderstedt, Germany) was used, which was stuck to the surface of the metallized paper, and then torn off in one smooth movement. The adhesive strength of the deposited copper layer was roughly determined on the basis of visual and microscopic evaluation of the tape detachment point and the tape surface on the adhesive side.

## 3. Results and Discussion

**Figure 2:**
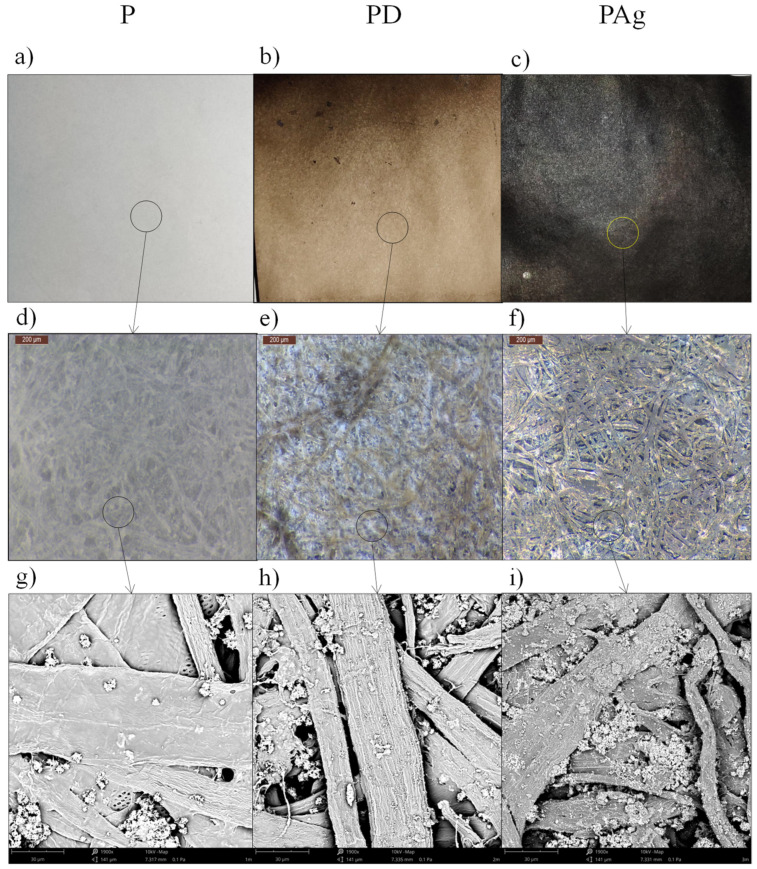
Macroscopic photos (first row), optical macroscopy (second row) and scanning electron microscopy (third row) of (**a**,**d**,**g**) blank paper (P), (**b**,**e**,**f**) paper with polydopamine coating (PD) and (**c**,**f**,**i**) paper after surface activation (PAg).

The surface of the unmodified paper was white, and the microscopic photos show the typical structure of interwoven cellulose fibers (Figure 2). The SEM images show that the surface of the cellulose fibers was relatively smooth with visible inclusions possibly being excipients used in papermaking. The composition of the surface layer was mainly composed of carbon (C) and oxygen (O) atoms (Table 1) which corresponds to the chemical structure of cellulose. In addition to the atoms of these two elements, small amounts of calcium (Ca) were also detected, which suggests that the inclusions visible in the SEM images were particles of chalk, a typical additive used in paper production.

After dipping the paper in the dopamine solution, both the macroscopic and microscopic appearance changed significantly. The first easily discernible effect was a color change. The white surface turned brown, which is typical of the deposition of a polydopamine layer. The color of this layer was brown, which is widely described in the literature [39,40,41]. The brown color of the cellulose fibers is also visible in the optical microscope images. The observed fibers became more visible as their edges and surfaces were colored brown. In addition, the SEM images show a significant change in the appearance of the surface of cellulose fibers The surface structure changed from smooth to ribbed, and the number of inclusions visible in the SEM photos increased significantly.

Dopamine, as the only compound used in the research, has nitrogen (N) in its structure. Therefore, based on the analysis of the quality and quantity of nitrogen atoms, it was possible to indirectly assess the effectiveness of the deposition process of the polydopamine coating. The appearance of nitrogen atoms (N) in the EDS analysis confirmed the formation of a polydopamine layer on the surface of cellulose fibers. The determined content of N atoms in the surface layer of the PD sample was 12.53 at.%, while the map of the distribution of this element shows that the polydopamine layer was deposited evenly on the entire modified surface of the paper (Figure 2).

The purpose of depositing the polydopamine layer was to use the ability of this layer to reduce metal ions and deposit silver (Ag) atoms on the surface of cellulose fibers. The deposited Ag was to serve as a catalyst for the process of electroless deposition of a copper layer. The paper with a layer of polydopamine was, therefore, dipped in an aqueous solution of silver nitrate (AgNO_3_). The effect of immersion was an almost instantaneous change of the color of the paper surface to black, which is a typical effect of reducing Ag^+^ ions to Ag^0^. The reduced Ag atoms were deposited on the surface of the cellulose fibers, changing the color of the PAg sample to black. This effect was visible not only on the macroscopic scale. The optical microscope photos also showed a darker color change of the cellulose fibers. Small changes in the structure of cellulose fibers were also observed in the SEM pictures, although they were not as visible as after the deposition of the polydopamine coating. Only the increased amount of visible particles, possibly aggregated Ag particles, deserves attention.

**Figure 3:**
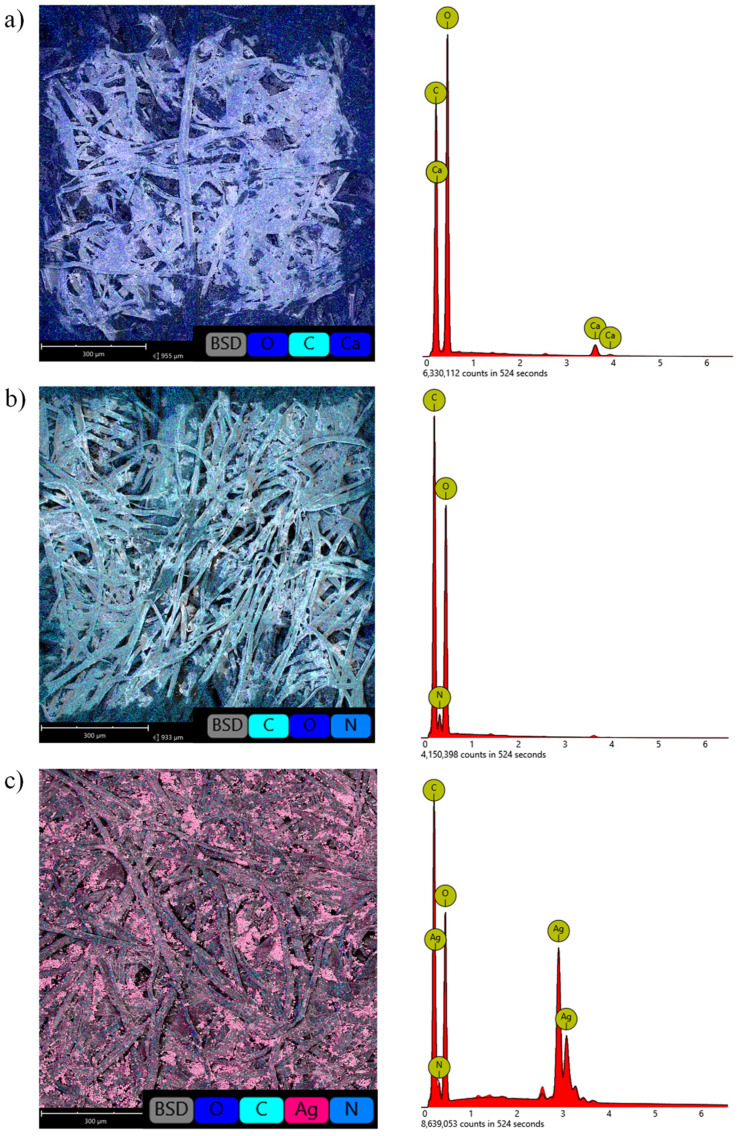
Results of EDS analysis of the surface layer of (**a**) P, (**b**) PD and (**c**) PAg samples.

The deposition of Ag atoms was confirmed by the EDS results. The analysis of the composition of the surface layer of the PAg sample showed the presence of this element on the paper surface. The content of Ag atoms in the surface layer was 7 at.% (Table 1). Silver particles were evenly distributed over the entire surface of the fibers, and also appeared in form of aggregates between the fibers (Figure 3).

**Figure 4:**
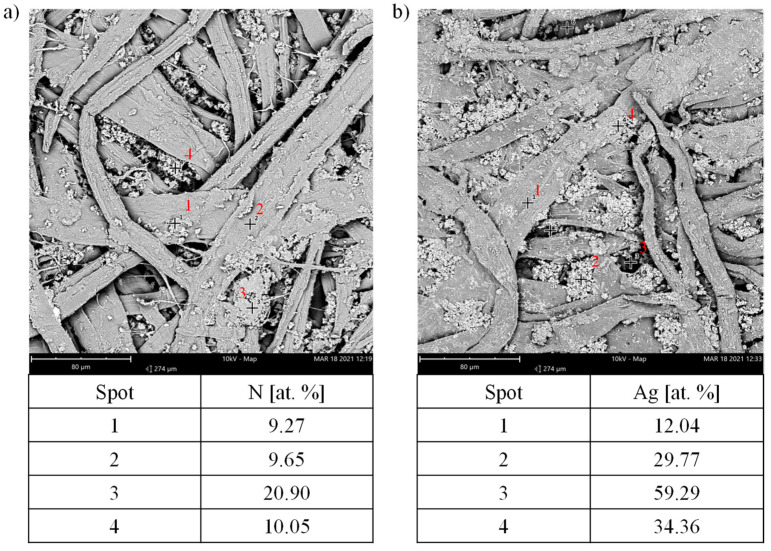
EDS surface point analysis of the (**a**) PD and (**b**) PAg samples.

In order to thoroughly analyze the effects of deposition of the polydopamine and Ag^+^ atoms, with particular emphasis on the exact distribution of these materials, EDS point analysis of PD and PAg samples was performed (Figure 4), focusing on the analysis of the amount of N and Ag atoms. The composition of the fiber surface and the visible aggregates, as well as the free spaces between the visible fibers, were analyzed.

The amounts of nitrogen atoms on the individual cellulose fibers of the PD sample (points 1 and 2) were similar (approx. 9 at.%), which proves that the fibers were evenly coated with polydopamine. It can be seen, however, that the polydopamine coating was not evenly deposited on the entire surface of the paper, as there were some differences in the amount of nitrogen deposited depending on the site of the analysis. Larger amounts of N atoms, up to 21 at.%, were found on the surface of the observed aggregates. It seems that a thick layer of polydopamine coating was also deposited on the chalk particles contained in the paper (point 3), confirming one of the most important features of polydopamine coatings, namely the ability to be deposited on virtually any type of substrate regardless of the material constituting it. The high content of N atoms in the cavities between the cellulose fibers (point 4) shows that the polydopamine coating could deposit not only in easily accessible places, but also easily reached deeper, less accessible places in the surface layer of the coated material. If the deposition of Ag atoms will be successful, this property may positively affect the adhesive strength of the finally deposited target metallic layer or the layer obtained by electroplating thickening. The possibility of embedding the metallic layer in the recesses of the surface will ensure good mechanical anchoring, which will result in an increase in adhesive strength.

The distribution of Ag atoms on the surface of the PAg sample deposited as a result of the reduction of Ag^+^ ions was more scattered than that of the N atoms in the PD sample. The EDS spot analysis showed that Ag atoms were deposited unevenly on the surface of the cellulose fibers. The content of Ag atoms on the surface of the fibers ranged from 12 to 34 at.% (points 1 and 4). On the other hand, larger amounts of Ag were deposited on the aggregates (point 2). It can be seen that the greater amount of polydopamine deposited on the chalk particles was the cause of a more intensive Ag^+^ reduction process, and thus, a greater amount of deposited Ag atoms. The analysis of the cavity between the cellulose fibers (point 3) showed that the reduction of Ag^+^ ions took place also in these hard-to-reach places. The content of Ag atoms in the analyzed point was very high (approx. 60%), which means that potential catalytic centers were also located in the cavities of the material’s surface layer. Consequently, the likelihood of high adhesion strength of the final copper layer increased according to the mechanism described in the previous paragraph.

**Figure 5:**
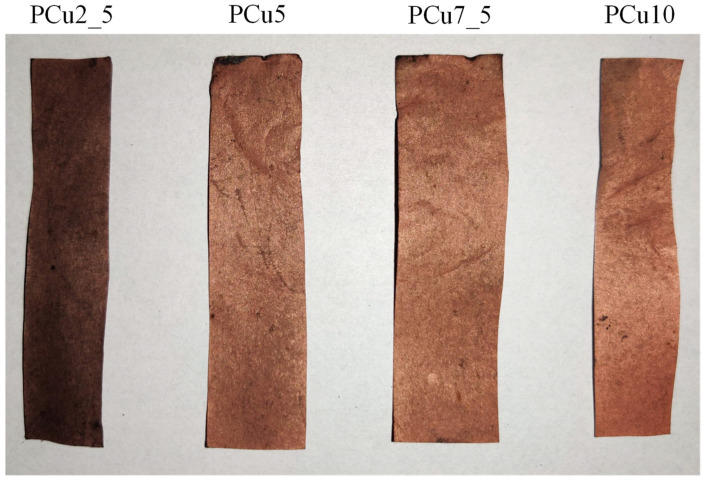
The effects of electroless copper plating of paper depending on the time of metallization.

In order to verify the possibility of metallizing the paper according to the proposed process, the samples were, after activation, immersed in a solution for electroless copper plating. The effects of the metallization process are shown in Figure 5.

As a result of metallization, a layer of metallic copper was deposited on the surface of the paper. After 2.5 min of metallization, the copper layer was barely visible, yet after 5 min in the metallization bath, the entire surface of the paper was covered with a clearly visible and homogeneous layer of copper. Further increasing the plating time no longer caused strong macroscopic changes and it was difficult to distinguish between materials with shorter and longer plating times.

**Figure 6:**
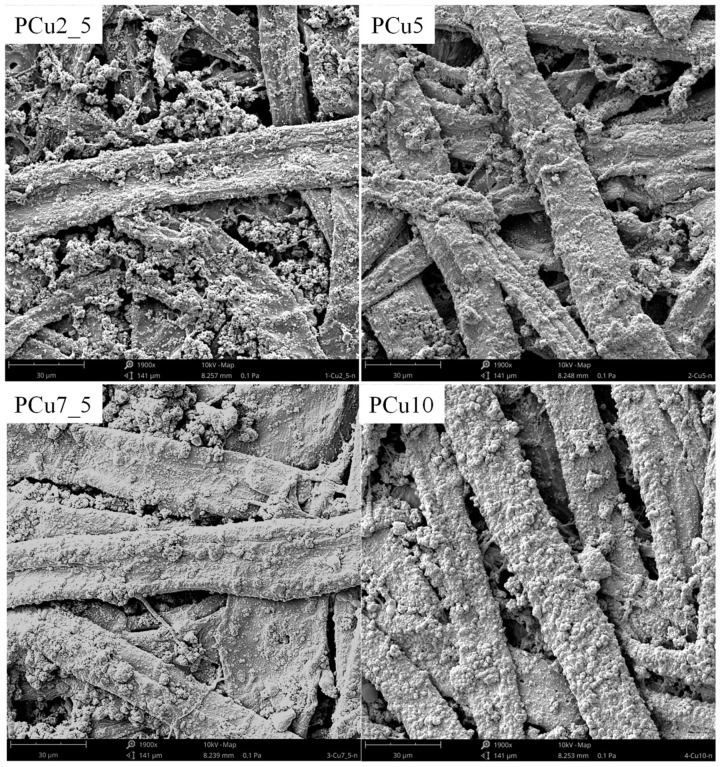
SEM surface images of metallized samples.

The differences in the structure and quantity of the deposited copper layer as a function of the metallization time are clearly visible in the SEM pictures (Figure 6) and in the results of the EDS analysis (Table 2).

The structure of the deposited copper layer initially had a granular structure, which is typical for metallic layers obtained by the autocatalytic electroless method. It is related that the active centers formed by Ag atoms, on which the process of reducing Cu^2+^ ions and depositing Cu^0^ take place, were distributed pointwise on the surface. Individual copper grains, therefore, grew independently of each other, forming the observed structure. Metallic copper grains are visible both on the cellulose fibers and spaces between them. The copper content on the surface of the PCu2_5 sample was approx. 32 at.%. Small amounts of N and Ag atoms could, however, still be detected in the surface layer.

After 7.5 min of metallization, the entire surface of the fibers was covered with a thick layer of copper. The results of the EDS analysis confirmed the increase in the amount and thickness of the deposited copper as the metallization time increased. The content of Cu atoms on the surface of the tested materials increased to approx. 56 at.%. for the PCu5 sample and approx. 84 at.% for sample PCu7_5. At the same time, the content of N and Ag atoms was clearly decreasing, which proved that the metallized surface was more and more thoroughly covered with a layer of copper, and that the thickness of this layer was increasing.

An interesting effect was observed for the PCu10 sample. As in the case of shorter metallization times, the entire surface of the fibers was thoroughly covered with a copper layer, but again we could observe a clear grain structure for the deposited layer. The protruding copper grains probably took over the function of active centers, influencing the re-formation of the grain structure. However, there was no significant increase in the content of Cu atoms on the surface of the material. The Cu content in the case of the PCu10 sample was about 88% at. There was also a slight decrease in the content of N and Ag compared to the PCu7_5 sample.

**Figure 7:**
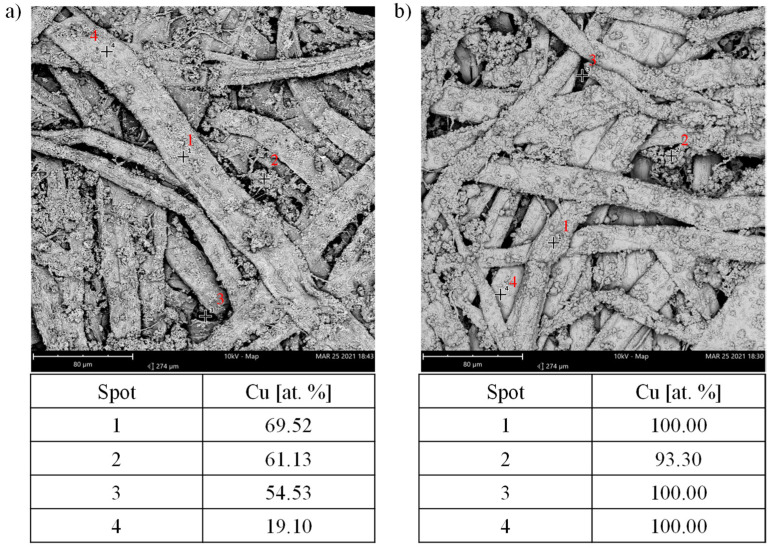
EDS surface point analysis of the (**a**) PCu2_5 and (**b**) PCu10 samples.

The EDS point analysis enabled a more accurate characterization of the deposition effects and distribution of the copper atoms. Figure 7 shows the results of the point analysis for the samples with the shortest and the longest metallization time.

In the case of the PCu2_5 sample, the distribution of Cu atoms on the surface of cellulose fibers was uneven and ranged from 19 at.%. (point 4) up to 69 at.% (point 1), which may have been the result of the previously observed uneven distribution of Ag atoms on the fiber surface in the PAg sample. The major advantage was that a significant amount of Cu atoms were also deposited in the cavities between the cellulose fibers. Thus, it can be seen that the proposed method of metallizing with the use of polydopamine coatings is perfect for metallizing surfaces with hard-to-reach places. 

An amount of Cu atoms above 90 at.% was observed in PCu10 sample. The copper content was very high regardless of the place of EDS analysis, confirming the very good surface coverage of the material.

The deposition of polydopamine, silver and then copper layer on the paper surface influenced the dielectric properties of this material. The values of surface resistivity and volume resistivity of selected samples are presented in Table 3.

The values of surface resistivity and volume resistivity of blank paper (P) were 1 × 10^13^ (Ω) and 1.8 × 10^11^ (Ωm), which was in line with literature values [42]. The deposition of the polydopamine coating increased the surface resistivity to 7.9 × 10^13^ (Ω), and the volume resistivity to 2.3 × 10^12^ (Ωm). The increase in the resistivity of the sample was due to the presence of a polymer coating on the surface of the material. In general, polymeric materials have surface and volume resistivity values of >10^14^. However, since the deposited polydopamine layer is very thin and does not completely cover the entire surface, the observed increase was not as large as might be expected.

The surface resistivity after deposition of silver atoms did not change significantly. The value of the PAg sample was 1.2 × 10^13^ (Ω). The lack of a significant decrease in surface resistivity, despite the deposition of conducting silver atoms, resulted from the arrangement of the deposited Ag atoms on the surface of the Pag sample. The silver atoms deposited in the surface activation process were rather loosely distributed over the entire surface of the material. Therefore, they did not form a homogeneous layer that was able to conduct the current efficiently and, therefore, lower the resistivity. On the other hand, the volume resistivity of the PAg sample decreased by two orders of magnitude to the value of 9.5 × 10^10^ (Ωm). Therefore, it is likely that the silver atoms were also deposited in the deeper layers of the material, and the three-dimensional structure created in this way increased the possibility of electric charges permeating through the material.

Significant changes in surface resistivity compared to pure cellulose paper were obtained after the metallization process. Depositing the copper layer for 10 min reduced the value of surface resistivity to 5.9 × 10^7^ (Ω), which is a value in the typical range for semiconductors. Taking into account the electrical properties of copper and the negligible value of the resistivity of this element, the obtained decrease in value was lower than expected. This fact was again caused by the granular deposition and growth of the applied metallic layers, which are characteristic for electroless metallization of non-conductive materials. With short deposition times, the resulting metal layer was not homogeneous, which made it difficult to conduct electricity. In addition, the characteristic structure of a sheet of paper, in which individual cellulose fibers have only point contact, also does not have a positive effect on the reduction in the surface resistivity despite the deposition of highly conductive copper atoms. It is likely that, with longer deposition times, the surface resistivity values would decrease as a function of time as a result of compacting the metal layer and filling the gaps between the cellulose fibers. Nevertheless, the obtained values of surface resistivity are sufficient for further processing of the material in the current process and obtaining a fully conductive material.

**Figure 8:**
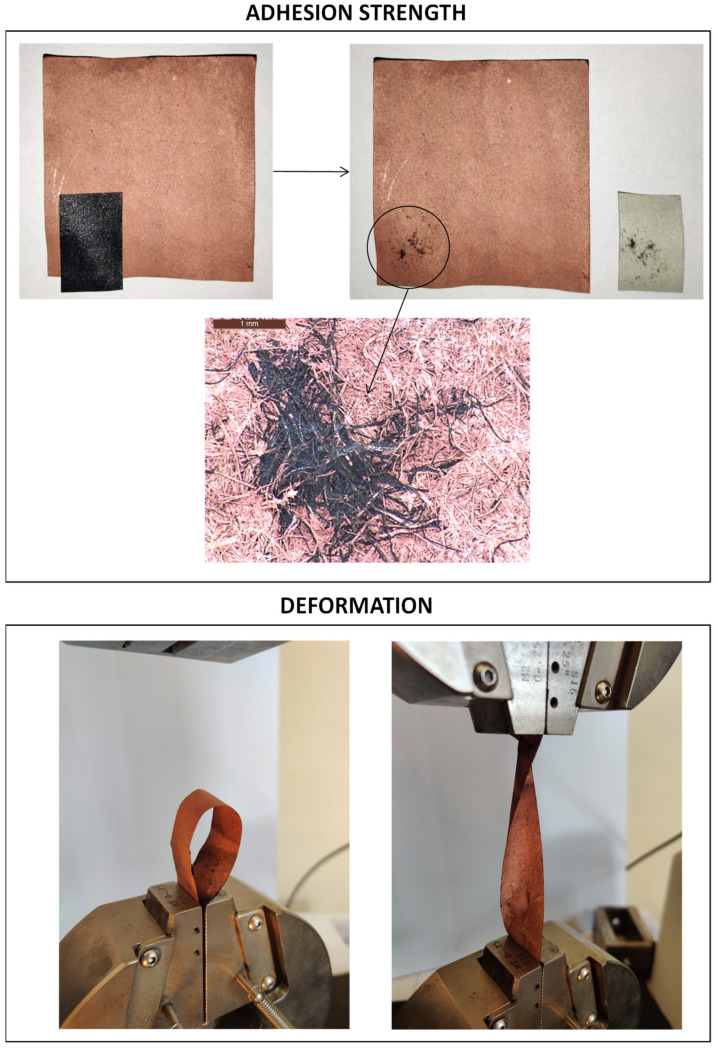
Adhesive strength and susceptibility to deformation of the deposited copper layer.

The tests of the adhesive strength of the deposited copper layer showed that it is characterized by very high adhesion to the surface of the paper sheet. After the adhesive tape was torn off, the surface was damaged; however, as shown by microscopic photos (Figure 8), this was related to the detachment of cellulose fibers from the paper volume, and not the detachment of the copper layer from cellulose fibers.

Additionally, the deposited copper layer showed excellent resistance to sheet deformation. Regardless of how the paper was deformed or bent with the deposited copper layer, this layer did not peel, crack or detach from the material surface. This is a necessary feature for systems to be used as flexible materials for electronic applications, an area where metallized cellulose paper is to be used.

## 4. Conclusions

The use of a polydopamine coating in the metallization of cellulose paper ended successfully with the deposition of a good quality copper layer. This process, however, required several steps. The first stage was the deposition of a polydopamine coating on the paper, the first and most easily noticeable effect of which was a change in the appearance of the paper. From white, the paper turned brown, which is a typical effect of applying a polydopamine coating to various materials. In addition to visual changes, the deposition of polydopamine coating was confirmed by scanning electron microscopy photos and energy dispersion spectroscopy analysis. A clear change in the surface structure of cellulose fibers was visible, along with the nitrogen signals in the analysis of the chemical composition of the surface, which confirmed the presence of polydopamine. Spot analysis showed that the polydopamine coating was evenly deposited on the surface of the paper, and the nitrogen content was between 9 and 21 at.%.

The unique properties of the polydopamine coating made it possible to reduce silver ions from an aqueous salt solution of this element and to deposit metallic silver atoms on the surface. The content of silver atoms on the surface of the paper ranged from 12 to 60 at.%. depending on where the chemical composition was analyzed.

Silver atoms successfully served as a catalyst for electroless metallization, as after 2.5 min of plating, a copper layer was visible on the surface of the paper, with a copper content of approx. 32 at.%. Increasing the metallization time increased the amount of deposited copper and, after 10 min, the copper content reached 88 at.%.

The deposition of the copper layer caused a large decrease in the surface resistivity of the paper from 10^13^ to 10^7^ (Ω), which corresponds to the order typical for semiconductors. Such resistivity will allow further current post-treatment of obtained electroless metallized paper. In addition, the obtained copper layer was characterized by high adhesive strength and high susceptibility to deformation, which will enable the use of the metallized cellulose paper in electronics, including flexible electronic devices.

## Figures and Tables

**Table 1:** EDS analysis of the composition of the surface layer of blank paper (P), paper with a polydopamine coating (PD) and paper after surface activation.

Sample	Element				
	C (at.%)	O (at.%)	N (at.%)	Ag (at.%)	Ca (at.%)
P	48.36	51.09	-	-	0.55
PD	46.48	40.99	12.53	-	-
PAg	42.70	43.44	6.86	7.00	-

**Table 2:** EDS analysis of the surface layer of metallized samples.

Sample	Element				
	C (at.%)	O (at.%)	N (at.%)	Ag (at.%)	Cu (at.%)
PCu2_5	30.92	26.72	5.79	5.46	32.22
PCu5	19.75	14.60	4.69	3.59	56.27
PCu7_5	6.18	7.35	0.64	1.51	84.33
PCu10	6.15	3.97	0.65	1.21	88.02

**Table 3:** Surface and volume resistivity of tested materials.

Sample	Surface Resistivity (Ω)	Volume Resistivity (Ωm)
P	1.0 × 10^13^	1.8 × 10^11^
PD	7.9 × 10^13^	2.3 × 10^12^
PAg	1.2 × 10^13^	9.5 × 10^10^
PCu10	5.9 × 10^7^	4.5 × 10^10^

## Data Availability

The data presented in this study are available on request from the corresponding author.
